# Effects of 1,25-Vitamin D3 and 24,25-Vitamin D3 on Corneal Nerve Regeneration in Diabetic Mice

**DOI:** 10.3390/biom13121754

**Published:** 2023-12-06

**Authors:** Xiaowen Lu, Zhong Chen, Jerry Lu, Mitchell A. Watsky

**Affiliations:** Department of Cellular Biology and Anatomy, Medical College of Georgia, Augusta University, 1120 15th Street, CB-2901, Augusta, GA 30912, USA

**Keywords:** vitamin D deficiency, cornea, nerve regeneration, corneal nerve fiber, wound healing, vitamin D receptor

## Abstract

Corneal nerve homeostasis is essential for the functional integrity of the ocular surface. Vitamin D deficiency (VDD) and vitamin D receptor knockout (VDR KO) have been found to reduce corneal nerve density in diabetic mice. This is the first study to comprehensively examine the influence of vitamin D on nerve regeneration following corneal epithelial injury in diabetic mice. Corneal nerve regeneration was significantly retarded by diabetes, VDR KO, and VDD, and it was accelerated following topical 1,25 Vit D and 24,25 Vit D administration. Furthermore, topical 1,25 Vit D and 24,25 Vit D increased nerve growth factor, glial cell line-derived neurotropic factor, and neurotropin-3 protein expression, and it increased secretion of GDNF protein from human corneal epithelial cells. CD45+ cells and macrophage numbers were significantly decreased, and vitamin D increased CD45+ cell and macrophage recruitment in these wounded diabetic mouse corneas. The accelerated nerve regeneration observed in these corneas following topical 1,25 Vit D and 24,25 Vit D administration may be related to the vitamin D-stimulated expression, secretion of neurotrophic factors, and recruitment of immune cells.

## 1. Introduction

The cornea is one of the most densely innervated tissues in the human body, containing 19,000 to 44,000 small nerve fiber afferents. It exhibits a complex nerve architecture, distribution, and structural organization [1]. The outermost layer of the cornea consists of a stratified squamous, non-keratinized epithelium. Cornea nerve fibers originate from cell bodies residing in the trigeminal ganglion, losing their myelin as they enter the cornea and terminating on the superficial surface of the cornea between the basal epithelium and Bowman’s layer. In addition to their important sensory functions, corneal nerves help to maintain epithelial homeostasis, which is essential for the functional integrity of the ocular surface [2]. Loss of corneal nerves in diabetic corneas and following corneal wounding, particularly in diabetic corneas, can contribute to diabetic keratitis and poor wound healing. 

Diabetic keratopathy, a condition that causes progressive damage to corneal epithelial cells and sensory nerves, affects up to 50% of patients with diabetes mellitus (DM) [3,4,5]. Nerve changes in diabetic corneas have been well described and include reduced nerve density, thin nerve fibers, and impaired nerve migration [6,7,8,9]. The degree of nerve loss increases with the duration of DM [10,11], and the accompanying reduction of neurotrophic stimuli can result in the thinning of the epithelial layer and recurrent corneal erosions [11]. The interactions between epithelium and intraepithelial nerves are critical for nerve regeneration and nerve maintenance. Only a few studies have examined nerve regeneration following epithelial injury in diabetic corneas. 

In addition to neuro–epithelial interactions, interactions between nerves and immune cells are also critical to corneal nerve maintenance and regeneration [12,13]. Resident immune cells distributed throughout the corneal epithelium and stroma include dendritic cells, macrophages, mast cells, and innate lymphoid cells [14,15]. Large numbers of CD45+ cells invade the stroma after mouse cornea epithelial debridement, including monocytes, macrophages, neutrophils, lymphocytes, and stem cells [16,17,18,19]. Stromal macrophages were found to promote nerve remodeling after epithelial abrasion [20] and to contribute to corneal homeostasis and pathology [21]. 

Vitamin D is both a hormone, as it can be synthesized from 7-dehydrocholestrol, and a vitamin, given that most populations do not synthesize enough vitamin D through the de novo pathway. Endogenous vitamin D is synthesized from 7-dehydrocholesterol in skin following UVB exposure, although vitamin D can also be activated and metabolized in the cornea [22]. The classical systemic route for vitamin D activation is initial hepatic 25-hydroxylation to 25-hydroxy vitamin D3, followed by renal conversion to the active 1,25-dihydroxy vitamin D3 (1,25 Vit D) by cytoplasmic 1α-hydroxylase. The conversion of 25(OH)D to 24,25-dihydroxy vitamin D3 (24,25 Vit D) is facilitated by 24(R)-hydroxylase, which is strongly induced by 1,25 Vit D [23,24]. 

Low serum vitamin D concentrations and serum vitamin D-binding protein levels have consistently been detected in patients with type 1 and type 2 diabetes mellitus (DM) [25,26,27], and there is a highly consistent inverse association between 25-hydroxy vitamin D concentration and incident diabetes [28]. Our group recently determined that corneal nerve density is significantly decreased in vitamin D-deficient diabetic mice (VDD) and vitamin D receptor knockout (VDR KO) diabetic mice [29]. Low corneal nerve density has also been documented in patients with DM [30]. In several neurodegenerative disorders, 1,25 Vit D has been shown to have neuroprotective effects, and VDR is present in neurons and glial cells [31,32]. Recognized for its positive effects on bone, 24,25 Vit D is active and likely physiologically significant in the corneal epithelium (in some cases more so than 1,25 Vit D) [33,34]. The current study examined the effects of vitamin D deficiency on nerve density in diabetic corneas. In addition, the expression and secretion of nerve growth factor (NGF), neurotropin-3 (NTF3), and glial cell line-derived neurotropic factor (GDNF) in corneal epithelial cells after exposure to 1,25 Vit D and 24,25 Vit D was measured. Corneal CD45+ cells and macrophage infiltration in diabetic mouse corneas following epithelial abrasion and subsequent treatment with topical 1,25 Vit D and 24,25 Vit D was also measured. Given the high prevalence of vitamin D deficiency and diabetes, the links between these two conditions and low nerve density in the cornea and other tissues, and the importance of cornea innervation to cornea function, it is important to determine if topical vitamin D treatment can positively influence reinnervation following corneal wounding. 

## 2. Material and Methods

### 2.1. Materials

The 1,25 Vit D and 24,25 Vit D included in our study were purchased from Enzo Life Sciences (Catalog # BML-DM200-0050, BML-DM300-0050, Farmingdale, NY, USA). Antibodies for β-tubilin and NGF were purchased from ABCAM (Catalog # ab18207, ab52918, Cambridge, MA, USA). GDNF and NTF3 antibody were purchased from ThermoFisher Scientific (Catalog # MA5-33142 and PA5-102315, Waltham, MA, USA). Pre-stained protein markers were obtained from Bio-Rad (Catalog # LC5699, Hercules, CA, USA). Polyvinylidene difluoride (PVDF) membrane and the enhanced chemiluminescence (ECL) detection system were obtained from Bio-Rad (Catalog # 1704272, 1705060). 

### 2.2. Animal Experiments

All animal studies were approved by the Augusta University IACUC, and the animals were treated according to the ARVO statement for the Use of Animals in Ophthalmic and Visual Research: https://www.arvo.org/About/policies/arvo-statement-for-the-use-of-animals-in-ophthalmic-and-vision-research/ (accessed on 30 November 2023). All the animals were housed in standard conditions with a 12 h dark–light cycle. The mice were euthanized via CO_2_ inhalation and cervical dislocation at the end of the experiments.

The low-dose streptozotocin (STZ) injection method was used to induce diabetes. Briefly, five sequential daily intraperitoneal injections of a freshly prepared solution of STZ in 0.1 mol/L citrate buffer (pH 4.5) at a dosage of 60 mg/kg body weight were administered to 4-week-old mice. Blood glucose was measured 1 week after the final STZ injection. We limited the mice receiving corneal epithelial wounds to those with blood glucose levels >249 and <651 mg/dL [35].

Male and female C57BL/6 mice were used in our studies. Table 1 lists all of the mouse groups used in this study along with their abbreviations. VDD mice were fed a vitamin D-deficient diet (TD.89123 Diet; Envigo, Tampa, FL, USA) for 4 weeks. The control group for the VDD mice was fed a TD.89124 vitamin D control diet (Envigo, Tampa, FL, USA). VDR KO Ca^++^ mice were fed a supplemental diet high in calcium, lactose, and phosphate (20% lactose, 2% calcium, 1.25% phosphate) (TD.96348 Diet; Envigo, Tampa, FL, USA) that has previously been shown to alleviate many of the VDR KO phenotypical features [36]. We previously documented serum 25-hydroxy vitamin D levels in diabetic and diabetic VDD mice and serum calcium levels in diabetic and diabetic VDD and VDR KO mice, along with VDR KO mice fed the supplemental diet [37].

### 2.3. Corneal Injury

Mice were anesthetized via intraperitoneal injection with a mixture of ketamine (200 mg/kg) and xylazine (50 mg/kg). A central section of the cornea was demarcated using a 2 mm trephine, and the epithelium and superficial anterior stroma were gently removed under a dissecting microscope using an Algerbrush II (Alger Equipment Co., Lago Vista, TX, USA) [38]. The mice were examined pre- and post-operatively to exclude ocular surface disease or excessive tissue removal, and they were removed from the study if inflammation or infection occurred. The mice were sacrificed and their eyes were enucleated 10 weeks after the corneal abrasion procedure to analyze their nerves.

### 2.4. Immunostaining for CD 45+ Cells and Macrophages

CD45+ cells and macrophages in the basal epithelium were immunostained after the corneas were wounded as described above and treated with 1,25 Vit D or 24,25 Vit D three times over 8 h. CD45 and F4/80 antibodies were used to immunolabel hematopoietic-derived immune cells and macrophages, respectively [19,39,40]. The eyes were fixed with Zamboni fixative (American MasterTech Scientific, Lodi, CA, USA) for 75 min and then washed three times with PBS. The corneas were carefully excised along the sclera–corneal border and subjected to a rehydration series with increasing concentrations of Triton X-100 in PBS. To block nonspecific binding, corneas were incubated with 10% normal goat serum with 0.1% Triton X-100 solution in PBS for 60 min at room temperature. The tissues were then incubated with primary anti-CD45 (Catalog# ab10558, Abcam, Cambridge, MA, USA), or anti-F4/80 (Catalog# ab18207; AbcamA) (1:200) antibody in PBS, containing 5% goat serum with 0.1% Triton X-100 for 24 h at 4 °C and constantly shaken. After washing with PBS three times for 10 min each, the corneas were incubated with the secondary antibody (1:1000) Alexa Fluor 594 goat anti-rabbit IgG (H + L) and Alexa Fluor 488 donkey anti-rat IgG (H + L) (Catalog# A-11012, A-21206, ThermoFisher Scientific, Norcross, GA, USA) for 24 h at 4 °C and washed thoroughly with PBS. Immunolabelled cells in the basal epithelial layer and macrophages in the stroma were quantified using the Imaris image processing software Spots package version 10.0.0 (Oxford Instruments, Abingdon, UK).

### 2.5. Vitamin D Administration and Treatment

The 1,25 Vit D and 24,25 Vit D were dissolved in 14.1 mM dimethyl sulfoxide (DMSO). The mice were treated with topical 40 nM 1,25 Vit D, 200 nM 24,25 Vit D, or with a vehicle control. The treatment consisted of one drop three times per day for 4 weeks, starting the day of corneal epithelial debridement. 

Human corneal epithelial cells from de-identified eye bank corneal rims and mouse primary corneal epithelial cells were isolated and cultured as previously described [41] and treated with 1,25 Vit D (20 nM) or 24,25 Vit D (100 nM). The control groups were treated only with the DMSO vehicle. The vitamin D treatment dosages were chosen based on previously measured vitamin D metabolite concentrations in the eye [22] and previous work from our laboratory using these dosages (e.g., [41]). 

### 2.6. Nerve Immunofluorescence Labeling and Imaging

Cornea nerve labeling and density measurements were made as previously described [8,29]. Briefly, the corneas were processed as described above for immunostaining. The corneas were then incubated with primary rabbit polyclonal anti-β III tubulin (1:500) antibody in PBS containing 5% goat serum with 0.1% Triton X-100 for 24 h at 4 °C. The secondary antibody was Alexa Fluor 488 goat anti-rabbit IgG (H + L) (Catalog# A-32731, ThermoFisher, Waltham, MA, USA). To image corneas, four radial cuts were made on each cornea, and the tissue was mounted flat on a slide with the epithelium side up. Images were taken using a Leica STELLARIS Confocal Microscope (Danaher Corporation, Washington, DC, USA).

To calculate nerve density, confocal images were taken just below the central cornea epithelium (four pictures relative to the center: left, right, top, bottom). The most central half of each photo was traced in ImageJ (version 1.53a). The average percentage of pixels traced, among the four central images, was defined as the nerve density value of the image [8,29].

### 2.7. Protein Extraction from Culture Medium

Human corneal epithelial cells were cultured with 1.5 mL DMEM medium (1% FBS) in a 35 mm dish (ThermoFisher Scientific, Catalog# 12-565-91), and were treated with 20 nM 1,25 Vit D or 100 nM 24,25 Vit D for 48 or 72 h. Cultured medium samples were collected and centrifuged at 800× *g* for 5 min. Trichloroacetic acid (TCA) was added at a final concentration of 12% to the post-centrifuged supernatants, and these were allowed to precipitate for at least 1 h on ice. The proteins were then pelleted via centrifugation at 16,000× *g* for 20 min at 4 °C and subsequently washed three times with cold acetone (4 °C). The samples were air dried and solubilized in lysis buffer (Cell Signaling Technology, Catalog# 2881S, Danvers, MA, USA) with protease inhibitor (Sigma, Catalog# P8340-1ML, Ann Arbor, MI, USA). Proteins were then characterized using Western blotting. 

### 2.8. Western Blot Analysis

Proteins were isolated from the culture medium or from human corneal epithelial cells grown on 35 mm dishes. The cultured cells were washed in PBS at 4 °C and treated with lysis buffer. Cell lysates were collected, and Western blotting was performed as previously described [34,42]. The membranes were incubated with primary antibodies of NGF (ABCAM, Catalog# ab52918, Cambridge, MA, USA), NTF3 and GDNF (ThermoFisher Scientific, Catalog# MA5-33142 and PA5-102315, Waltham, MA, USA) at a dilution of 1:1000 in Tris-buffered saline with 0.1% Tween 20 (TBST) and 10% non-fat milk at 4 °C overnight. Removal of excess primary antibody was carried out by washing the membranes in TBST three times for 10 min each. The secondary goat anti-rabbit IgG antibody (H + L) (ThermoFisher Scientific, Catalog# 31460) was diluted 1:3000 and incubated with the membrane in TBST with 10% non-fat dry milk for one 1 hr at room temperature. Detection and quantification of band intensities was conducted using Image Lab 5.2.1 software (Bio-Rad). The bands were normalized by dividing the intensity of the band by the intensity of the total protein from the same sample on the blot using the Bio-Rad Stain-Free method, as we have previously described [34]. The Western blot data are provided as the mean ± SE of at least three experiments.

### 2.9. Statistical Analysis

Comparisons between multiple groups were analyzed using an ANOVA. Data comparisons between only 2 groups were analyzed using a student’s unpaired *t*-test comparing experimental groups against controls. *p* < 0.05 was considered statistically significant. GraphPad Prism 8.4.3 was used for the data analysis (GraphPad Software, La Jolla, CA, USA).

## 3. Results

### 3.1. Diabetic Nerve Regeneration

**Figure 1:**
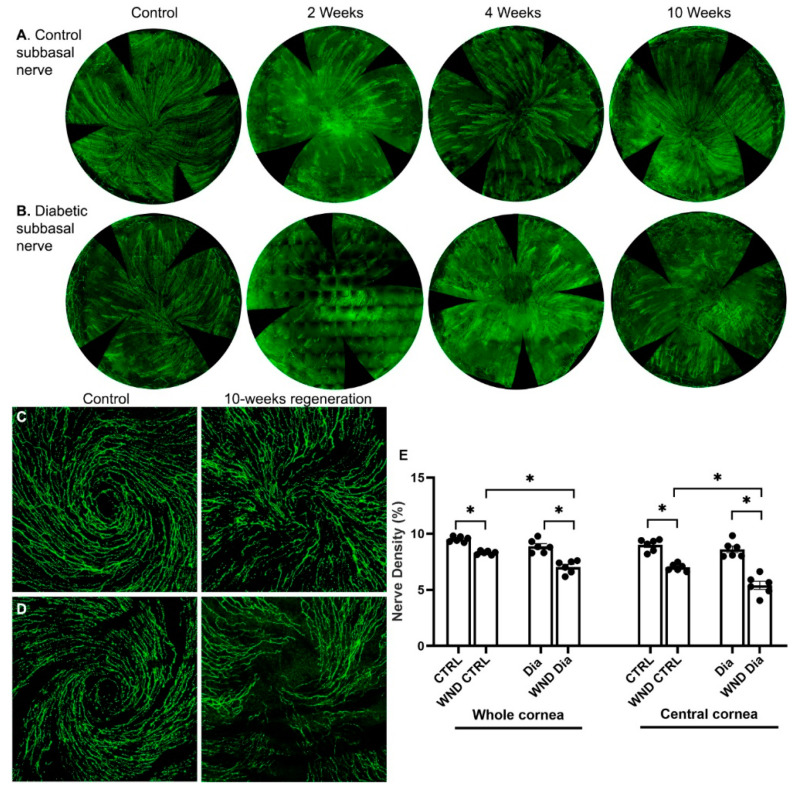
Nerve regeneration in control and diabetic mice. (**A**) Representative sub-basal whole cornea nerve regeneration in (**A**) control and (**B**) diabetic mice 2, 4, and 10 weeks after corneal abrasion. (**C**,**D**) Representative sub-basal nerve regeneration in the central cornea of control (**C**) and diabetic mice (**D**) 10 weeks after corneal abrasion. (**E**) Sub-basal nerve density quantification of whole and central corneas of control and diabetic mice 10 weeks following corneal abrasion. Individual data points graphed as mean ± SE, *n* = 6, * *p* < 0.05.

Sub-basal nerves were lost following epithelial cell and superficial stroma removal. After 2 weeks, clusters of regenerated sub-basal nerves were observed in wounded normoglycemic and diabetic corneas (Figure 1A,B). In normoglycemic mice at 4 weeks, the sub-basal nerves were more developed, and 10 weeks after wounding, a developing whorl-like pattern was observed (Figure 1C). In diabetic mice, the sub-basal intraepithelial nerves regenerated more slowly, and the whorl-like pattern was only partially developed (Figure 1B,D). Sub-basal nerve density over the whole cornea and separately in the central cornea was measured 10 weeks after wounding (Figure 1E). The WND CTRL whole cornea nerve density was significantly lower than CTRL (8.3 ± 0.16% vs. 9.5 ± 0.23%, *p* < 0.05), and the WND Dia corneas had a significantly lower density than the Dia corneas (7.0 ± 0.57% vs. 8.9 ± 0.58%, respectively). The WND Dia whole cornea nerve density was significantly lower than the WND CTRL density. 

Limiting the density measurement to the central cornea (Figure 1E), which regenerates more slowly the peripheral cornea, 10 weeks after wounding, the WND CTRL central cornea nerve density was significantly lower than the CTRL (7.0 ± 0.32% vs. 9.0 ± 0.53%), and the nerve density of the WND Dia central corneas was significantly lower than the Dia central corneas (5.4 ± 0.88% vs. 8.6 ± 0.72%). The central density of the WND Dia corneas was also significantly lower than that for the WND CTRL corneas. 

### 3.2. Diabetic Vitamin D Deficiency Nerve Regeneration

**Figure 2:**
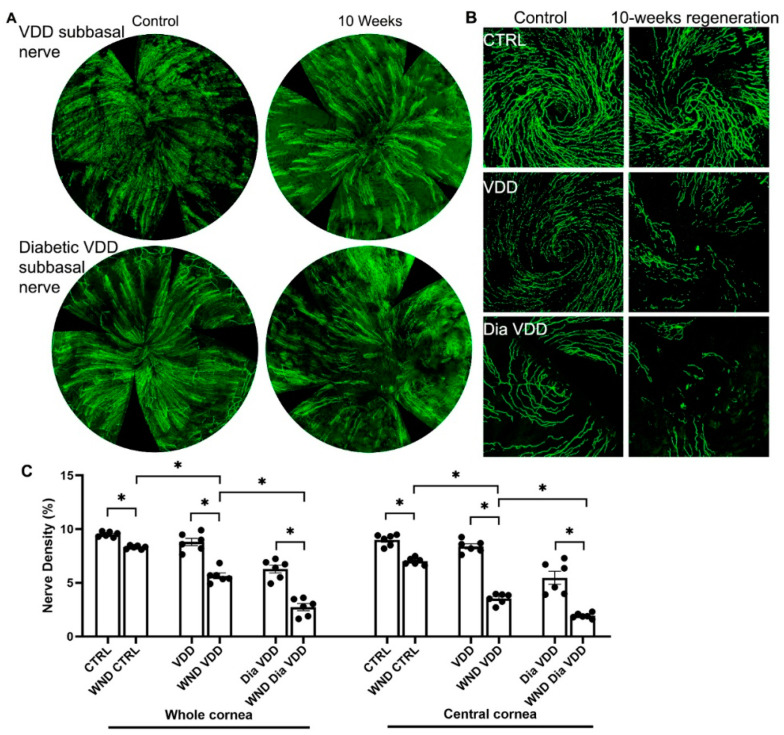
Nerve regeneration in VDD and diabetic VDD mice. (**A**) Representative sub-basal whole cornea nerve regeneration in VDD and diabetic VDD mice 10 weeks after corneal abrasion. (**B**) Representative sub-basal nerve regeneration in the central cornea of VDD and diabetic VDD mice 10 weeks after corneal abrasion. (**C**) Sub-basal nerve density quantification of whole and central corneas of VDD and diabetic VDD mouse corneas 10 weeks following corneal abrasion. Individual data points graphed as mean ± SE, *n* = 6, * *p* < 0.05.

10 weeks after wounding, the whorl-like pattern of the sub-basal nerve plexus did not develop in the VDD or Dia VDD mice (Figure 2A,B). Figure 2C shows whole and central cornea nerve density quantification for the VDD groups. The WND VDD whole cornea nerve density was significantly lower than the VDD nerve density (5.6 ± 0.65% vs. 8.8 ± 0.83%, respectively), and was also lower than the WND CTRL density (*p* < 0.05). The WND Dia VDD mouse corneal nerve density was significantly lower than the Dia VDD density (2.7 ± 0.81% vs. 6.3 ± 0.88%, respectively), and it was also significantly lower than the WND VDD density. Similar significant changes were observed when examining the central cornea nerve densities of the same VDD groups.

### 3.3. Diabetic VDR KO and VDD Nerve Regeneration

**Figure 3:**
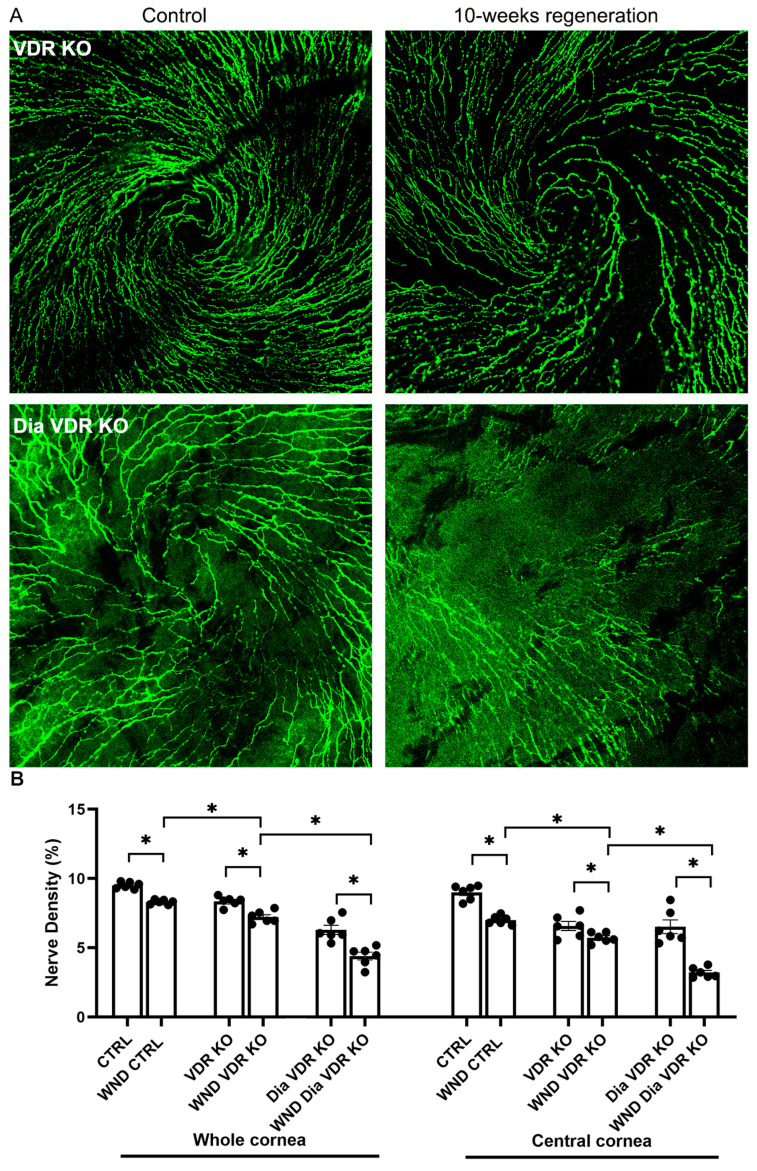
Nerve regeneration in VDR KO and diabetic VDR KO mice. (**A**) Representative sub-basal nerve regeneration in the central cornea of VDR KO and diabetic VDR KO mice 10 weeks after corneal abrasion. (**B**) Sub-basal nerve density quantification of VDR KO and diabetic VDR KO mouse corneas 10 weeks following corneal abrasion. Individual data points graphed as mean ± SE, *n* = 6, * *p* < 0.05.

Initiation of the sub-basal nerve plexus whorl-like pattern was observed in VDR KO mouse corneas but not in the Dia VDR KO mouse corneas 10 weeks after wounding (Figure 3A). Figure 3B shows whole and central cornea nerve density quantification for the VDR KO groups. The whole cornea sub-basal nerve density was significantly lower in the WND VDR KO mice vs. the WND CNTRL and VDR KO mice (7.2 ± 0.43% vs. 8.3 ± 0.16% vs. 8.4 ± 0.36%, respectively), and the WND Dia VDR KO nerve density was significantly lower than that of the Dia VDR KO mice (4.4 ± 0.69% vs. 6.3 ± 0.81%, respectively). The WND Dia VDR KO density was also significantly lower than the WND VDR KO density. There was no significant difference between the whole cornea WT and VDR KO nerve densities; however, the WND VDR KO mice had significantly lower nerve densities compared with WND CTRL mice. Similar significant changes were observed when examining central cornea nerve densities. Comparing the densities of the Dia WT mice (from Figure 1E) with the Dia VDR KO mice, the Dia VDR KO mice had significantly lower nerve densities (4.4 ± 0.69% vs. 7.0 ± 0.57%, respectively). The Dia VDD mouse nerve density was lower than that of the Dia VDR KO mice, although the difference was not significant (*p* = 0.07).

### 3.4. Nerve Regeneration in Dia VDR KO Mice Fed the Supplemental Diet

**Figure 4:**
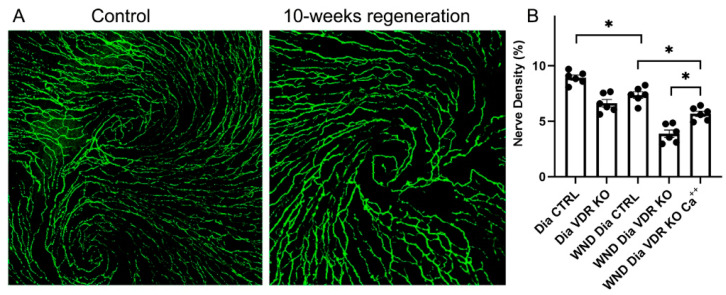
Dia VDR KO mice with central corneal abrasions that were fed the supplemental diet significantly increased whole corneal nerve density, but not to control levels. (**A**) Representative sub-basal nerves in the central cornea of unwounded (left) and wounded (right) diabetic VDR KO mice fed the supplemental diet for 10 weeks. (**B**) Nerve density measurements. Individual data points graphed as mean ± SE, *n* = 6, * *p* < 0.05.

Corneas were wounded in the Dia VDR KO mice, which were then fed a supplemental diet enriched in Ca^++^ to determine if reductions in serum Ca^++^ brought on by VDR KO is linked to changes in nerve regeneration. The whole cornea nerve density was increased in diabetic VDR KO mice fed the supplemental diet for 10 weeks after wounding, although not to unwounded levels (Figure 4A,B). Interestingly, unlike the Dia VDR KO mice fed the routine diet, the sub-basal nerve whorl-like pattern was partially restored in the wounded diabetic VDR KO mice fed the supplemental diet (Figure 4A compared to Figure 3A (bottom right)). 

### 3.5. Nerve Regeneration in Wounded Normoglycemic Mouse Corneas Following Topical Vit D Treatment

**Figure 5:**
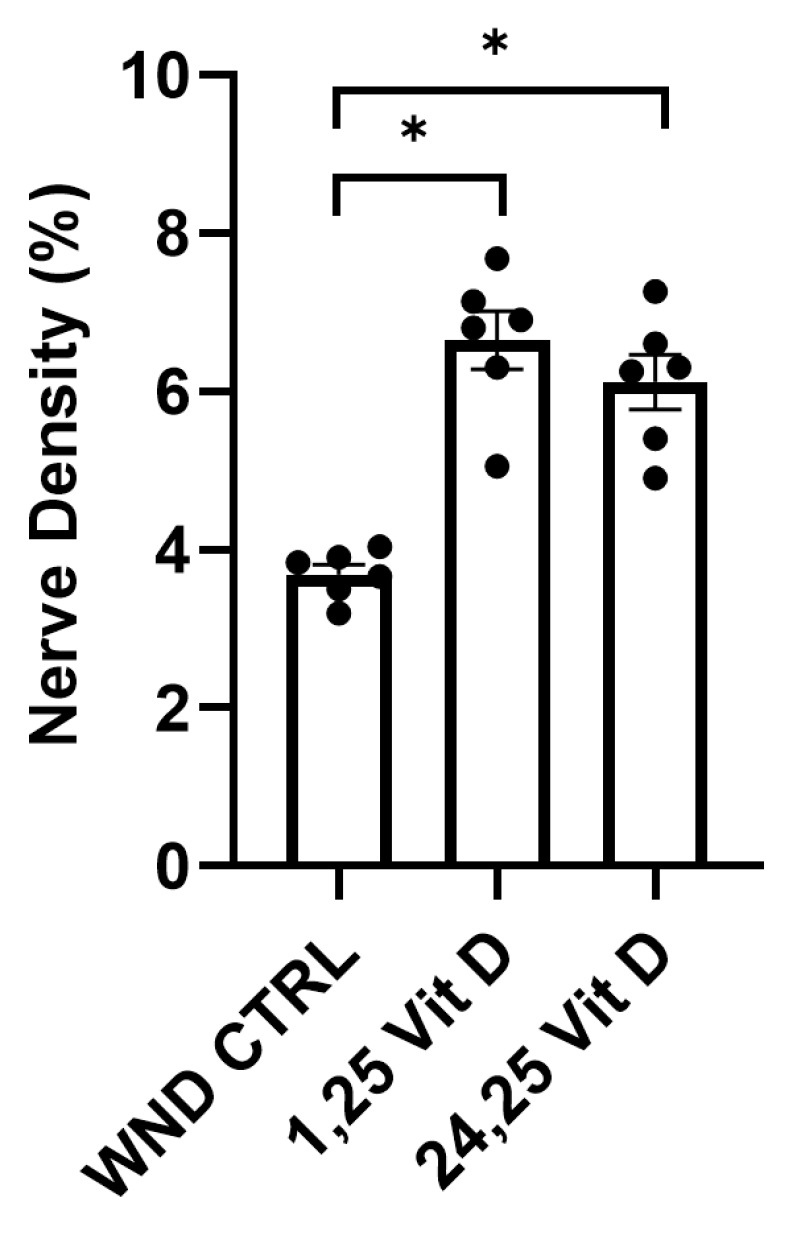
Topical vitamin D3 increases whole corneal nerve regeneration in control mouse corneas following epithelial abrasion. Nerve density measurements of normoglycemic mouse corneas treated with 40 nM 1,25 Vit D or 200 nM 24,25 Vit D for 4 weeks. Individual data points graphed as mean ± SE, *n* = 6, * *p* < 0.05.

The wounded mouse corneas were treated with 1,25 Vit D or 24,25 Vit D drops 3 times daily for 4 weeks following epithelial abrasion. Whole cornea sub-basal nerve density was significantly higher in the 1,25 Vit D and 24,25 Vit D-treated mice vs. the WND CTRL mice (6.6 ± 0.89% vs. 6.1 ± 0.84% vs. 3.7 ± 0.31%, respectively) (Figure 5).

### 3.6. Nerve Regeneration in Diabetic and Dia VDD Mouse Corneas Following Topical Vit D Treatment

**Figure 6:**
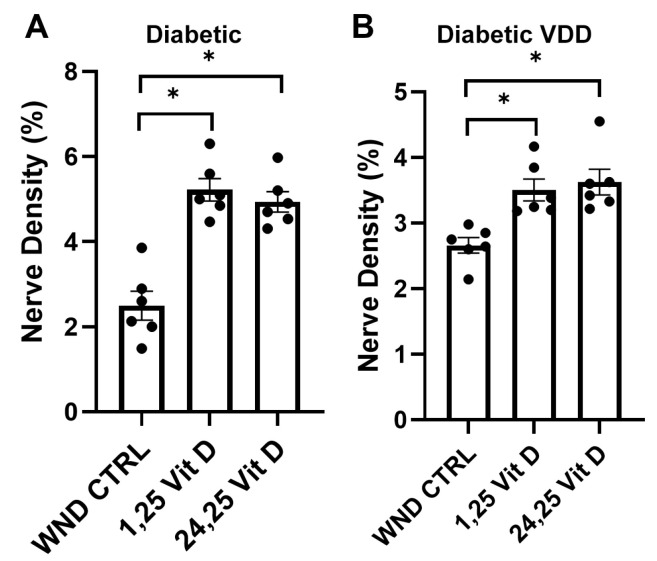
Topical vitamin D3 increases whole corneal nerve regeneration in diabetic and diabetic VDD mouse corneas following epithelial abrasion. Nerve density measurements of (**A**) diabetic and (**B**) diabetic VDD mouse corneas treated with 40 nM 1,25 Vit D or 200 nM 24,25 Vit D for 4 weeks. Individual data points graphed as mean ± SE, *n* = 6, * *p* < 0.05.

Topical 1,25 Vit D application significantly enhanced whole-cornea sub-basal reinnervation in the WND Dia and WND Dia VDD mice compared to the untreated WND Dia and WND Dia VDD mice (5.2 ± 0.64% vs. 2.5 ± 0.82% and 3.5 ± 0.4% vs. 2.6 ± 0.28%, respectively) (Figure 6). Topical 24,25 Vit D application also significantly increased reinnervation in the WND Dia and WND Dia VDD mice (4.9 ± 0.59% vs. 2.5 ± 0.82%, 3.6 ± 0.48% vs. 2.6 ± 0.28%, respectively) (Figure 6). 

### 3.7. Effects of 1,25 Vit D and 24,25 Vit D on NGF Protein Expression

**Figure 7:**
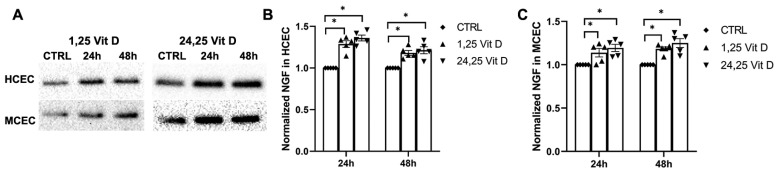
1,25 Vit D and 24,25 Vit D increase NGF protein expression in human and mouse primary cornea epithelium. (**A**) Representative Western blots from primary epithelial cells treated with 1,25 and 24,25 Vit D. (**B**,**C**) Corresponding average blot density graphs with total protein normalization from (**B**) human and (**C**) mouse primary corneal epithelial cells treated with 1,25 and 24,25 Vit D. Individual data points graphed as mean ± SE, *n* = 5, * *p* < 0.05. Points at value of 1.0 are normalized controls. Original images for blots are shown in Supplementary Figure S1.

NGF protein expression was increased in the cultured human corneal epithelial cells (HCECs) and the mouse corneal epithelial cells (MCECs) 24 h and 48 h after 1,25 Vit D and 24,25 Vit D exposure (Figure 7; *p* < 0.05).

### 3.8. Effects of 1,25 Vit D and 24,25 Vit D on NTF3 Protein Expression

**Figure 8:**
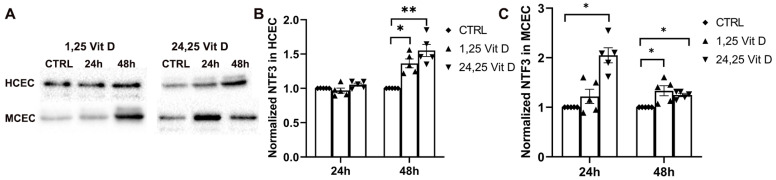
1,25 Vit D and 24,25 Vit D increase NTF3 protein expression in human and mouse primary cornea epithelium. (**A**) Representative Western blots from primary epithelial cells treated with 1,25 and 24,25 Vit D. (**B**,**C**) Corresponding average blot density graphs with total protein normalization from (**B**) human and (**C**) mouse primary corneal epithelial cells treated with 1,25 and 24,25 Vit D. Individual data points graphed as mean ± SE, *n* = 5, * *p* < 0.05, ** < 0.01. Points at value of 1.0 are normalized controls. Original images for blots are shown in Supplementary Figure S2.

NTF3 protein expression was significantly increased in the HCECs 48 h after 1,25 Vit D and 24,25 Vit D exposure (*p* < 0.05 and *p* < 0.01, respectively), but not at 24 h. MCEC NTF3 protein expression was significantly increased 24 h after 24,25 Vit D exposure and 48 h after 1,25 Vit D and 24,25 Vit D exposure (Figure 8; *p* < 0.05).

### 3.9. Effects of 1,25 Vit D and 24,25 Vit D on GDNF Protein Expression

**Figure 9:**
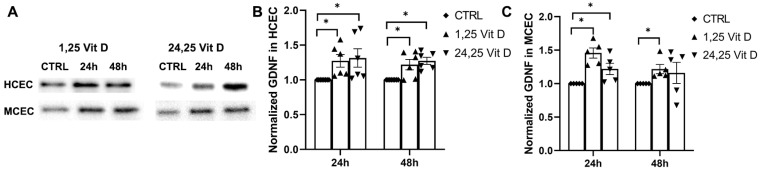
1,25 Vit D and 24,25 Vit D increase GDNF protein expression in human and mouse primary cornea epithelium. (**A**) Representative Western blots from primary epithelial cells treated with 1,25 and 24,25 Vit D. (**B**,**C**) Corresponding average blot density graphs with total protein normalization from (**B**) human and (**C**) mouse primary corneal epithelial cells treated with 1,25 and 24,25 Vit D. Individual data points graphed as mean ± SE, *n* = 5, * *p* < 0.05. Points at value of 1.0 are normalized controls. Original images for blots are shown in Supplementary Figure S3.

HCEC GDNF protein expression was significantly increased 24 h and 48 h after 1,25 Vit D and 24,25 Vit D exposure. MCEC GDNF protein expression was significantly increased by 1,25 Vit D and 24,25 Vit D after 24 h (*p* < 0.05), and by 1,25 Vit D after 48 h exposure (Figure 9). 

### 3.10. Effects of 1,25 Vit D and 24,25 Vit D on HCEC NGF, NTF3, and GDNF Protein Secretion

**Figure 10:**
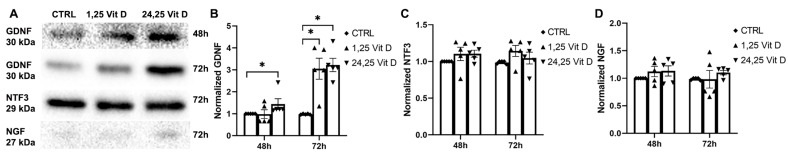
1,25 and 24,25 Vit D increase GDNF but not NGF and NTF3 protein secretion in HCECs. (**A**) Representative GDNF, NTF3, and NGF Western blots from conditioned medium of HCECs treated with 1,25 and 24,25 Vit D. (**B**–**D**) Corresponding average blot density graphs with total protein normalization for (**B**) GDNF, (**C**) NTF3, and (**D**) NGF from HCECs treated with 1,25 and 24,25 Vit D. Individual data points graphed as mean ± SE, *n* = 5, * *p* < 0.05. Points at values of 1.0 are normalized controls. Original images for blots are shown in Supplementary Figure S4.

Baseline NGF, NTF3, and GDNF secretion into the medium of the cultured HCECs was observed. GDNF secretion was significantly increased in the HCECs exposed to 24,25 Vit D at 48 and 72 h (Figure 10B) and following 1,25 Vit D exposure at 72 h. NGF and NTF3 secretion was not significantly increased in the HCECs treated with 1,25 Vit D or 24,25 Vit D (Figure 10C,D).

### 3.11. CD45+ Cell Recruitment Following 1,25 Vit D and 24,25 Vit D Treatment

**Figure 11:**
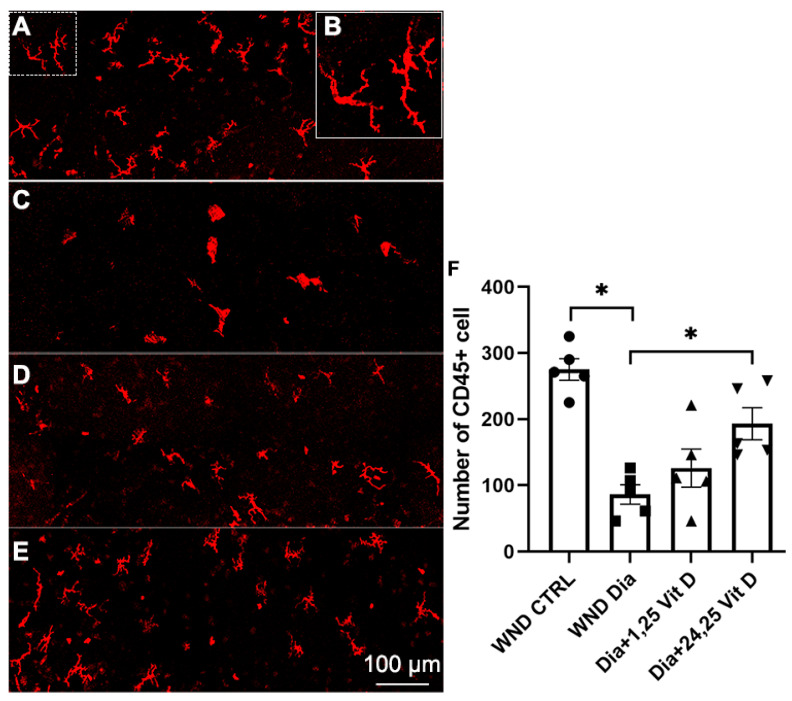
Representative dendritic cells labeling (CD45+) in the basal epithelium of (**A**) control, (**C**) diabetic, (**D**) diabetic plus 1,25 Vit D, (**E**) diabetic plus 24,25 Vit D corneas, and (**F**) quantification of cell numbers for each group in (**A**–**E**). (**B**) Magnified image of dendritic-shaped cells from the dashed area in the upper left of (**A**). The diabetic corneas had fewer dendritic cells than the normoglycemic corneas following cornea abrasion, and topical 24,25 Vit D significantly increased the number of dendritic cells in the diabetic corneas. Individual data points graphed as mean ± SE, *n* = 5, * *p* < 0.05.

Hematopoietic-derived immune cells in the basal epithelium were detected using CD45 immunostaining 8 h after wounding of the diabetic mouse corneas (10 weeks diabetic duration). Figure 11 illustrates significantly reduced CD45+ cell numbers in the basal epithelium of diabetic (Figure 11C) vs. normoglycemic mice (Figure 11A). When treated with topical 24,25 Vit D, CD45+ cells numbers were significantly increased in the basal epithelium of the diabetic mice (Figure 11E,F), with no significant change following 1,25 Vit D treatment (Figure 11D,E). Most of the cells had a dendriform morphology, as illustrated in (Figure 11B).

### 3.12. Macrophage Recruitment Following 1,25 Vit D and 24,25 Vit D Treatment

**Figure 12:**
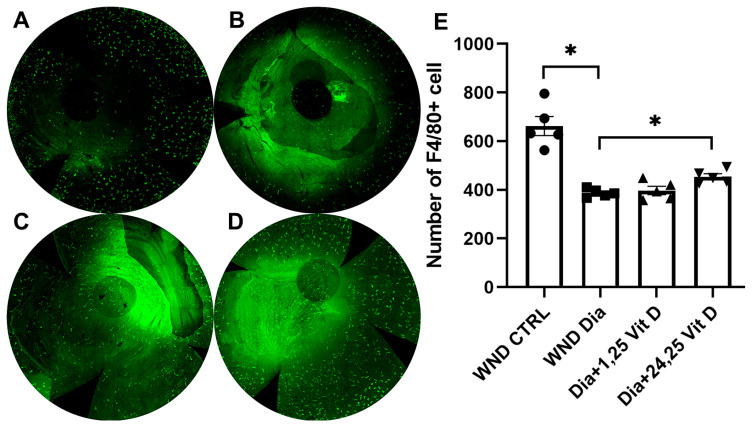
Representative macrophage labeling (F4/80) in the anterior stroma of (**A**) control, (**B**) diabetic, (**C**) diabetic plus 1,25 Vit D, and (**D**) diabetic plus 24,25 Vit D corneas. (**E**) The diabetic corneas had fewer macrophages than the normoglycemic corneas following cornea abrasion, and topical 24,25 Vit D significantly increased the number of macrophages in the diabetic corneas. Individual data points graphed as mean ± SE, *n* = 5, * *p* < 0.05.

Macrophages in the anterior stroma were identified using F4/80 immunostaining 8 h after wounding of the mouse corneas (10 weeks diabetic duration). Figure 12 illustrates significantly reduced macrophage numbers in the anterior stroma of the diabetic (Figure 12B) vs. normoglycemic mice (Figure 12A). When treated with topical 24,25 Vit D, the macrophage numbers were significantly increased in the anterior stroma of these diabetic mice, although not to pre-treatment levels (Figure 12D,E), with no significant change following 1,25 Vit D treatment (Figure 12C,E). 

## 4. Discussion

Previous studies have found that corneal sensation recovery following laser in situ keratomileusis begins within 4 weeks, with preoperative levels reached after 6 months in the majority of studies [43,44]. Full nerve density regeneration (as opposed to sensation) can take up to 12 or 24 months [45]. The effects of vitamin D on the nervous system have been studied extensively for the past two decades, leading to an accumulation of evidence regarding its roles in the regulation of neurotropic factor synthesis and its neuro-protective actions [46]. However, few papers have explored the effects of vitamin D3 on corneal nerve regeneration, and few commercial eye vitamin formulations include vitamin D3.

Vitamin D3 deficiency is common in diabetic patients. Corneal nerve fiber density, branch density, and nerve fiber length have been shown to be significantly different in diabetic patients relative to controls [3,13]. Previous studies in diabetic mice have determined that the density of sensory nerve fibers was significantly decreased compared to normoglycemic mice [9,13], and a recent report from our group determined that uninjured diabetic VDD and VDR knockout mouse corneas have significantly decreased nerve densities [29]. The current study demonstrates slower corneal nerve regeneration following corneal injury in diabetic mice compared to normoglycemic mice. We also found that vitamin D3 deficiency and VDR KO slow nerve regeneration in both diabetic and normoglycemic mice. 

The current study determined that nerve regeneration following corneal injury in diabetic VDR KO mice was increased by the high calcium supplemental diet, although not to WT levels. This is consistent with our previous work demonstrating that unwounded diabetic VDR KO mouse corneas have lower nerve densities than wild-type diabetic mouse corneas, with the same high calcium diet partially restoring this nerve density, but not to the diabetic wild-type value [29]. This indicates that the reduced nerve regeneration observed in the wounded VDR KO mice is likley associated with, but not entirely due to, hypocalcemia induced by VDR KO.

It is generally believed that 24,25 Vit D is an inactive form of vitamin D3. However, previous work from our laboratory has demonstrated that 24,25 Vit D stimulates HCEC proliferation and migration [41]. We also found that 24,25 Vit D is a positive regulator of connexin proteins and gap junction communication in the corneal epithelium [37]. In addition, 24,25 Vit D increases plectin and integrin β4 protein expression in mouse corneas and positively influences cornea desmosome and hemidesmosome junction formation/regulation [33]. Furthermore, in corneal fibroblasts, 24,25 Vit D was found to increase expression of the vitamin D-associated metabolic enzymes 24-hydroxylase and 1α-hydroxylase in the absence VDR KO mice, and thus is likely involved in fibroblast vitamin D metabolism regulation independent of 1,25 Vit D or VDR [34]. The current study adds to the list of potential benefits of 24,25 Vit D for corneal health, demonstrating that nerve regeneration is significantly increased in control and diabetic VDD mice treated topically with 24,25 Vit D. 

Complementing the nerve regeneration data and the positive influence of 24,25 Vit D on corneal function, this study determined that 1,25 Vit D and 24,25 Vit D both increase NGF, NTF3, and GDNF protein expression in mouse and human corneal epithelial cells, although GDNF was the only factor found to have its secretion level increased by Vit D (both 1,25 and 24,25). This is in agreement with previous studies showing 1,25 Vit D to be a potent inducer of NGF, GDNF, and NTF3 protein expression [47,48]. In addition, expression levels of NGF and GDNF were found to be reduced in VDD newborn rats [49], and gene expression of NGF is reduced in the brains of VDD rats [50,51]. These results indicate that 1,25 Vit D and 24,25 Vit D-induced synthesis of endogenous neurotropic factors can, at least in part, explain the observed vitamin D stimulation of corneal nerve regeneration following corneal injury. 

Previous studies have found that nerve growth factor expression is reduced in the skin and muscle of diabetic patients [52,53]. In addition, a recent study in a diabetic rat model found that oral administration of a vitamin D3 derivative, CB1093, acted as a highly potent inducer of NGF gene expression that prevented depletion of nerve growth factor and the products of its neuronal target genes [54]. It is possible that vitamin D has the potential to act similarly in the cornea of diabetic patients.

Vitamin D has been shown to be an immunomodulatory agent, exerting immunologic activities on multiple components of the innate and adaptive immune system [55]. The current study found that CD45+ cells with a dendriform morphology were significantly decreased in the basal epithelial layer of wounded diabetic mouse corneas compared to normoglycemic mice, with topical 24,25 Vit D increasing their numbers. These results are in agreement with an earlier study from our laboratory in which the corneas received only an epithelial abrasion with no stromal damage, and cells were counted at the level of the highest cell density, as opposed to the basal epithelium, where corneal nerves terminate [19]. Pertinant to the current study, it has been shown that CD45+ cells and macrophages play a pivotal role in the mechanisms involved in peripheral nerve regeneration and degeneration [56]. It is interesting that 24,25 Vit D increased the number of CD45+ cells in the diabetic mice corneas and increased nerve regeneration 8 h after wounding. While not examined in this study, it has been postulated that immune cells can mediate corneal nerve innervation and regeneration through ciliary neurotrophic factor release from immune cells in the cornea [9]. 

The current study also found a significant decrease in the number of F4/80+ cells in diabetic corneas compared to normoglycemic mouse corneas, with 24,25 Vit D increasing F4/80+ cell numbers in the diabetic mouse corneas 8 h after wounding. Although there are some exceptions, F4/80+ cells in the cornea are generally considered to be macrophages. Cornea macrophages reside throughout the anterior and posterior regions of the corneal stroma [57]. Stromal macrophages contribute to corneal homeostasis and pathology [58], although data demonstrating their precise role in normal corneal homeostasis are still rare [15]. Relevant to this study, corneal macrophages have been shown to promote nerve remodelling after epithelial abrasion [20]. In addition, it was previously determined that macrophages can increase corneal nerve regeneration by stimulating the secretion of neurotrophins, including nerve growth factor (NGF) and neurotrophin 3 (NTF3) [59]. 

This study has a number of limitations. Western blotting, a semi-quantitative method, was used to measure proteins. To bring this more into the quantitative realm, protein levels were quantified using total protein normalization rather than quantification using a single a housekeeping protein. Total protein quantification takes into account the intensities of all proteins in the lane, sample loading variations, variations during electrophoresis, and variations that occur during transfer. This resolves the inherent difficulties with linearity in the immunodetection of both target and control proteins, resulting in more accurate quantification of the target protein [60]. In addition, while measuring CD45+ and F4/80+ immune cell infiltration in the different mouse models describes the infiltration of two different classes of immune cells, it does not describe specific immune cell type infiltration.

## 5. Conclusions

This study found that nerve regeneration following corneal injury is significantly delayed by diabetes, VDD, and VDR KO, with compounded delays found in diabetic VDD and VDR KO mice. In addition, nerve regeneration in the VDR KO mice is partially calcium-dependent. Corneal sub-basal nerve regeneration in diabetic and VDD mice was significantly increased following administration of topical 1,25 Vit D and 24,25 Vit D. Topical vitamin D also increased NGF, NTFS, and GDNF corneal epithelial cell protein expression along with secretion of GDNF. Post-injury, CD45+ and F4/80+ cell numbers were significantly decreased 8 h after wounding, and 24,25 Vit D significantly increased the numbers of both cell types, although not to unwounded levels. This increase in immune cell numbers along with the increased expression of neurotrophic factors may contribute to the neural regenerative properties of vitamin D. This study demonstrates the positive role of vitamin D in corneal nerve regeneration and the potential of exogenous vitamin D treatment to improve corneal wound healing outcomes. 

## Figures and Tables

**Table 1:** Mouse groups and their abbreviations.

Mouse Groups		Abbreviation
Normoglycemic	Wild type	CTRL
	Vit D receptor knockout	VDR KO
	Vit D receptor knockout on supplemental diet	VDR KO Ca^++^
	Vitamin D deficient	VDD
Diabetic	Wild type	Dia CTRL
	Vit D receptor knockout	Dia VDR KO
	Vit D receptor knockout on supplemental diet	Dia VDR KO Ca^++^
	Vitamin D deficient	Dia VDD

WND abbreviation added in front of wounded groups.

## Data Availability

Data will be made available from the corresponding authors upon request.

## Supplementary Materials

The following supporting information can be downloaded at: https://www.mdpi.com/article/10.3390/biom13121754/s1, Figure S1: Uncropped images of Figure 7 western blots; Figure S2: Uncropped images of Figure 8 western blots; Figure S3: Uncropped images of Figure 9 western blots; Figure S4: Uncropped images of Figure 10 western blot.
