# Supplementary material for: Effects of 1,25-Vitamin D3 and 24,25-Vitamin D3 on Corneal Nerve Regeneration in Diabetic Mice
Source: Biomolecules. 2023 Dec 6;13(12):1754. doi: 10.3390/biom13121754 (PMC10742127; doi:10.3390/biom13121754)
Supplement: Supplementary file 1 [file biomolecules-13-01754-s001.zip › biomolecules-2734252-supplementary.pdf]

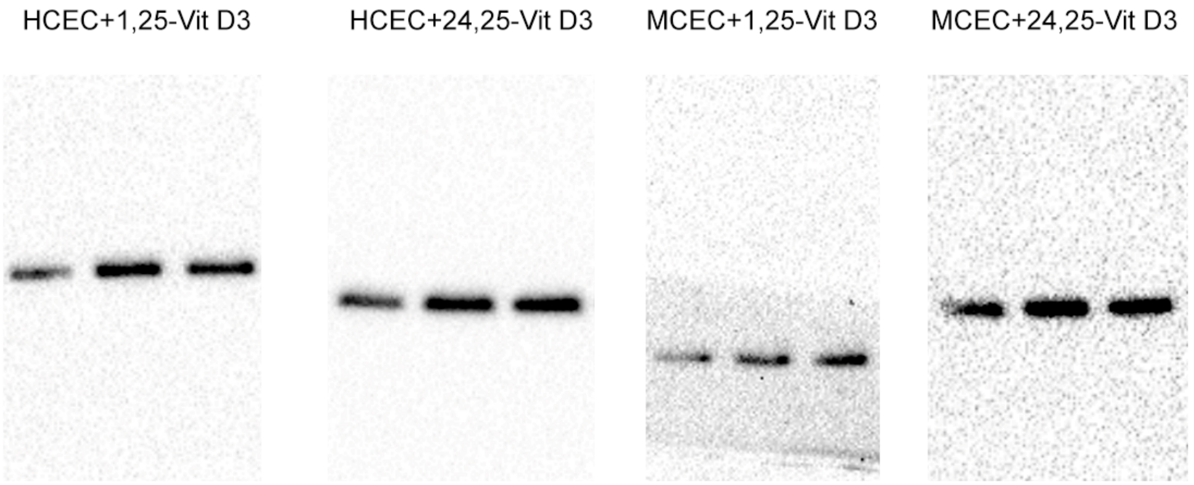

Figure S1. Uncropped images of Figure 7 western blots.

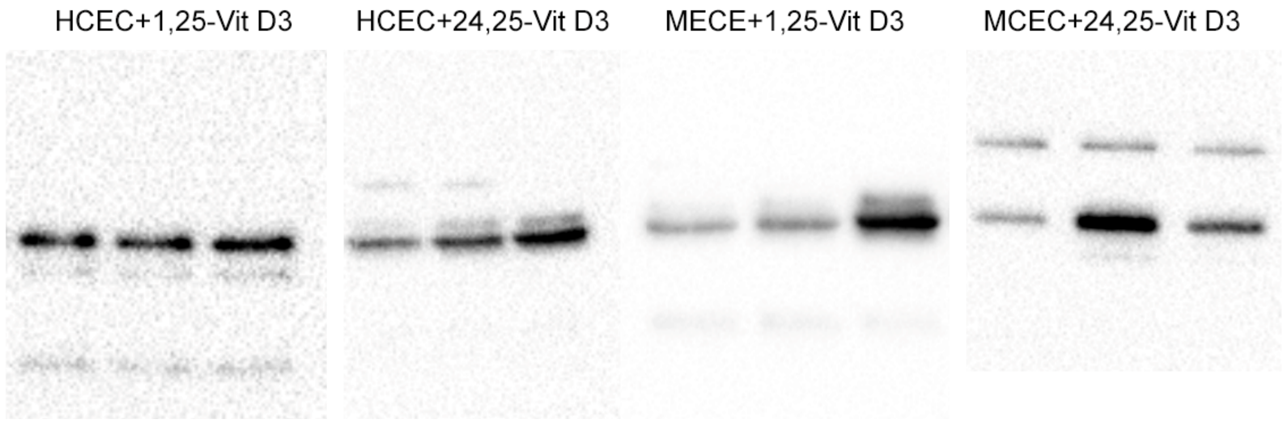

Figure S2. Uncropped images of Figure 8 western blots.

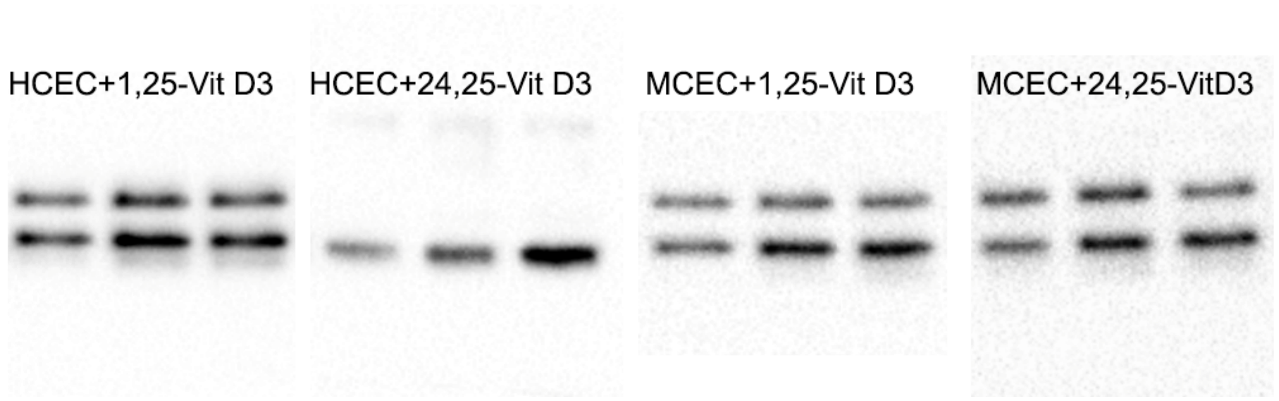

Figure S3. Uncropped images of Figure 9 western blots.

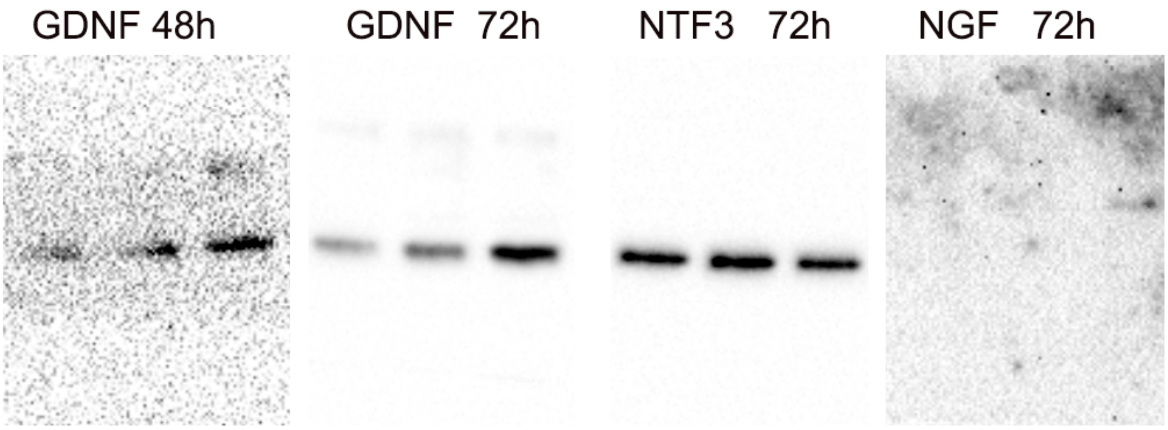

Figure S4. Uncropped images of Figure 10 western blot.
